# A Modified H-index Calculated Using the Duration of Professional Experience and the Author's Significant Contribution to Publications

**DOI:** 10.7759/cureus.53274

**Published:** 2024-01-31

**Authors:** Faruk Tas, Kayhan Erturk

**Affiliations:** 1 Medical Oncology, Institute of Oncology, Istanbul University, Istanbul, TUR; 2 Medical Oncology, Koç University, Istanbul, TUR

**Keywords:** citation metrics, research and publication, scientific performance, professional experience, author contribution, modified, h-index

## Abstract

Recently, the h-index (HI) has been accepted as a globally valid indicator that measures the professional achievement of scientists. Although it has been criticized in some respects, it is the most frequently used parameter as a criterion that generally measures the number and quality of publications combined. In the evaluation of the scientific publication, apart from these, the duration (years) of professional experience of the scientist and the impact/significance of the author's contribution to publications should also be used; however, HI does not take these into account. In this article, we present our recommendations for a modified HI considering these two important parameters.

## Introduction

Today, the h-index (HI) is the most frequently used tool in the scientific arena to evaluate the performance of authors [1]. As a numerical indicator, it shows the success of the scientist's productivity both qualitatively and quantitatively. The increasing citation rates with the increasing number of publications show the scientific success of the scientist with the increasing HI value. Although HI has aspects to be criticized, for example, the value of very high citation numbers is constrained by a small number of publications, it continues to be the most frequently used evaluation indicator today [2].

HI is not considered to optimally evaluate the scientific achievements of scientists, and it is a fact that it ignores some important parameters [2]. The most noteworthy impediments of HI are that it disregards the years of professional publishing experience and ignores the impact/significance of the author's contribution to the published articles. We examine these two significant matters in detail and present our suggestions in this report.

## Technical report

Duration of professional life

The length of professional experience of a scientist is proportional to the number of articles published by him/her. Naturally, the number of times these articles are cited will be influenced greatly by this duration. The longer a scientist works, the more articles will be published and, in turn, the more will be the number of citations to these publications. For example, let’s take a look at two authors with the same HI, but one of them has a professional experience of 10 years and the other 40 years. In our opinion, it is unfair to the former author with only one-fourth of the years of experience spent in the scientific field to be graded equally as the latter author, as it is done according to HI value. So, we believe that it will be more appropriate that the HI value should be modified by the duration of previous professional experience. Our suggestion for determination of the length of the author's professional life is as follows: the time between the year of his/her first publication and the year of HI evaluation/calculation (or the year of death, if the author is deceased). Then, the result will be divided by 100, and then 1 will be added to it; finally, it will be placed as the denominator of the fraction with the HI value being the numerator. This fraction will create the new HI-value, modified HI (mHI), by the length of professional productivity. Now, why don’t we test our new method? Let's say the HI value of the authors we mentioned earlier is 20 for each.

Professional life duration (PLD)=Year of HI calculation-Year of the first publication

mHI value based on PLD for the first author (10 years of experience) = HI/(1+PLD/100)

= HI/(1+[year of HI calculation-year of the first publication]/100)

=20/[1+10/100]

=20/[1+0.1]

=20/1.1

=18.18

mHI value based on PLD for the second author (40 years of experience)= 20/[1+40/100]

=20/[1+0.4]

=20/1.4

=14.29

We strongly believe that, in regard to scientific productivity, an author with a long professional experience should not be evaluated as equal to another author with a shorter career duration just because their HI values are the same. We suggest that our more appropriately and correctly calculated mHI values much better reflect, appreciate, and approbate the achievements of these authors based on their professional tenure.

Contribution of the author to a publication

The inability to assess the author's contribution to the production of the article is another important deficiency of the HI. Undoubtedly, all of the authors must have contributed to some degree to the publication, yet it should also be considered that the impact/significance of the contribution of each of these authors cannot be equal. Naturally, the most important contributor is the first-named author, and it would be unfair to rank him/her on the same level as the other authors. Even though not as much as the first author, we think that the other important position is the last author. Apart from this, the contribution of the author will be at the maximum level in single-authored publications. Therefore, the necessity of rewarding these three responsible persons is unquestionable. For example, let's assume that one of the two authors with the same HI is the first name in most of his/her articles, and the best position of the second author is the third name. It is beyond doubt that, in terms of scientific productivity, these two authors are not of equal value. How we suggest that the first name, single name, and last name in a publication be extra-rewarded is as follows:

The percentage of the first name publications and the percentage of single name publications multiplied by 2 and half of the percentage of the last name publications are added; then, the sum should be divided by 100 and 1 is added to the fraction, and the result is multiplied by HI. Thus, the mHI value created as per the author's place in the publication is reached. Let’s test our new method with an example: an author with an HI of 20 is the first name in 40% of his/her publications, a single name in 20%, and the last name in 10%; on the other hand, the other author does not have either a single name or first name publication, and he/she is the last name in 10% of his/her publications.

Author contribution (AC) = first name publication (%) + 2xsingle name publication (%) + last name publication (%)/2

mHI of first author based on AC = (1+[AC(%)/100])xHI

= (1+[first name publication(%)+2xsingle name publication+1/2xlast name publication]/100)xHI

mHI of the first author based on AC will be =(1+[40%+2x20%+1/2x10%]/100)x20

=(1+[40%+40%+5%]/100)x20

=(1+[85/100])x20

=(1+0.85)x20

=1.85x20

=37

mHI of second author based on AC = (1+[2x0%+0%+1/2x10%]/100)x20

=(1+[5/100])x20

=(1+0.05)x20

=1.05x20

=21

We believe that, regarding scientific productivity, the author with primary responsibility for many of his/her publications should not be held as equal to another author who contributed less. We suggest that our new mHI value differentiates the degree of achievements of these authors much better based on their contribution levels to the publication.

Calculation of mHI based on the duration of professional experience and author contribution

One problem that arises from our new suggestions is that the separate calculations of mHI, based on the duration of professional experience and the author's contribution will be inefficient and impractical. Thus, we had to find a way to unite these two separate evaluations. As a result, the following new calculation ensued: the decrease in the HI value resulting from extended professional tenure will be compensated by the increase in the level/impact of the author's contribution. Let's calculate one more time the mHI values of the two authors mentioned above.

mHI=HIx[1+(first name(%)+2xsingle name(%)+1/2last name)/100]/[1+(year of the calculation-year of the first publication)/100]

The first author's mHI: 

=(37/18.18)xHI

=(37/18.18)x20

=2.035x20

=40.7

The second author’s mHI: 

=(21/14.29)x20

=29.39

The mHI values determined by the levels/impacts of the contribution of the authors with different professional tenures will theoretically be between half and three times the HI values.

HI/2<mHI<3HI

To better understand the subject, we compared the HI and mHI value changes of four imaginary authors with different qualifications in Table 1. The differences in the authors’ scientific productivities/strengths are demonstrated accurately and more appropriately.

**Table 1:** Examples of mHI and HI changes and differences according to author characteristics *time period (years) = year of the HI evaluation/calculation - year of the first publication; **contribution (%) = the sum of first (%), 2xsingle (%), and last name publications (%)/2 HI: h-index; mHI: modified h-index

Nature of the author	Hard worker and highly qualified	Lazy and highly qualified	Hard worker and low qualified	Lazy and low qualified
Productivity of the author	Short time period* (10 years) + High contribution** (40%)	Long time period (40 years) + High contribution (40%)	Short time period (10 years) + Little contribution (10%)	Long time period (40 years) + Little contribution (10%)
HI	20	20	20	20
mHI	25.5	20	20	15.7
Difference, %	+27.5	0	0	-21.5

## Discussion

Previous reports, in which the professional achievements of scientists are listed on a large scale and other parameters apart from the HI values ​​of the authors were evaluated, also questioned the inadequacies that we discussed above [3-5]. These parameters were composed of: the year of the first publication, the year of the most recent publication, the number of single name publications, the total number of citations to single name publications, the number of single+first name publications, the total number of citations to single+first name publications, number of single+first+last name publications, and the total number of citations to single+first+last name publications.

While the methods we suggested above can be applied to all publications of the author, we believe it will be more rational and practical to apply them to scientifically valuable publications. Thus, we suggest that the best publications that generate the HI value, on whichever platform the author's HI is determined (such as Web of Science (WoS), Scopus, Google Scholar, Researchgate, etc), be evaluated. For example, let's suppose that the author has a HI of 20 in WoS; then the 20 publications with the highest citation numbers generating HI should be taken into consideration. Then, the parameters we suggest above (percentages of single, first, and last name publications, and the time difference between the evaluation/calculation and the oldest publication) should be determined in these publications and put into the formula, and the mHI value will be calculated according to that particular platform.

## Conclusions

The HI, still the most important indicator of scientific power/performance/productivity is far from ideal because of its inadequacy/inefficaciousness per se. We strongly believe that new formulas and modifications incorporating into the calculations the length of professional time of the author and the level/impact of the author's contribution are in great need. In our opinion, an author with a shorter professional experience and/or with primary responsibility for many of his/her publications should not be evaluated as equal to another author with a longer career duration who contributes less, just because their HI values are the same. We suggest that our more appropriately calculated mHI values much better reflect, appreciate, and approbate the achievements of these authors based on both their professional tenure and contribution to the publication. We believe that our suggestion is of great significance, and this modified index we propose will lead to many future studies in this direction.
